# What is the adsorption efficiency and capacity of biodegradable polymer blend foams filled with carbonaceous materials in the removal of heavy metals from water? A systematic review protocol

**DOI:** 10.1186/s13750-026-00384-x

**Published:** 2026-03-29

**Authors:** Lesia Sydney Mokoena, Pennie Petrus Mokolokolo, Julia Puseletso Mofokeng

**Affiliations:** Department of Chemistry, University of the Free State (QwaQwa campus), Private Bag X13, Phuthaditjhaba, 9866 South Africa

**Keywords:** Biodegradable polymer blend foams, Carbonaceous materials, Heavy metals, Contamination, Water remediation, Systematic review, Adsorption capacity

## Abstract

**Background:**

Water contamination by heavy metals poses serious risks to human health, ecosystems, and food security. Established and commonly used remediation methods have limitations in terms of cost, sustainability, and environmental impact, especially in under-resourced, developing, and underdeveloped countries. Recently, the emergence of biodegradable polymer blend foams filled with carbonaceous materials, such as graphene oxide and carbon nanotubes, offers environmentally friendly adsorbents for heavy metals in water. However, a consolidated, synthetic overview of the evidence on material design, properties, adsorption capacities, and efficiencies remains lacking for these foams. The current systematic review aims to synthesise and critically evaluate the existing evidence on the fabrication, properties, adsorption mechanisms, and applicability of biodegradable polymer blend foams filled with carbonaceous materials for adsorbing heavy metals in water. The review also seeks to develop a consolidated evidence document that will inform policy, guide future research, and assist the development and implementation of sustainable treatment technologies for the removal of heavy metals in water in resource-limited areas.

**Methods:**

This systematic review protocol is based on the question, “What is the adsorption efficiency and capacity of biodegradable polymer blend foams filled with carbonaceous materials in the removal of heavy metals from water?” A comprehensive search will be conducted using electronic bibliographic sources like Web of Science (core collections), Scopus, ScienceDirect, and search engines like PubMed, together with grey literature like Google Scholar, targeting peer-reviewed studies on laboratory and pilot-scale investigations of biodegradable polymer blend foams filled with carbonaceous materials for the removal of heavy metals from water. Eligibility criteria include studies on any type of water contaminated with heavy metals such as lead, copper, chromium, and cadmium. The extraction of data will be focused on material composition, processing techniques, characterisation, and adsorption performance. Quantitative data will be tabulated and, where applicable, subjected to meta-analysis. Researchers, consultants, and municipalities - especially in South Africa- will be consulted to contextualise findings and assist in translating evidence into practical water treatment solutions.

**Supplementary Information:**

The online version contains supplementary material available at 10.1186/s13750-026-00384-x.

## Background

The contamination of water by heavy metals remains a global concern, with serious health and environmental effects on humans, animals, and plants [1, 2]. Heavy metals are generally defined as metals with a density that is higher than 5 g.cm^− 3^, and common examples include lead (Pb), copper (Cu), chromium (Cr), and cadmium (Cd) [2]. These metals originate from natural sources such as rocks, soil, and volcanoes, as well as anthropogenic sources such as mining, various industries, and agriculture [3]. Waste from industries, as well as natural processes such as erosion, usually deposits these heavy metals in the ground, where they are transported by wind, gravity, and surface runoff to water bodies [3]. Once in water bodies, these non-biodegradable metals are difficult to remove due to their persistence and bioaccumulation [1–4]. Although various technologies have been developed and applied to remove these metals from water, the effectiveness of these techniques is still questionable in terms of costs, environmental impact, and sustainability [4–6]. The contaminated water eventually makes its way to drinking and agricultural water, especially in developing countries and under developing countries where there are poor economic capabilities to effect clean water and sanitation. As a public health and safety issue and in line with the United Nation’s sustainability goals 12, 13 and 14 [7, 8], it is imperative that the strides made in developing environmentally friendly and sustainable water treatment technologies are explored and built upon.

Of the various technologies that have been previously and continuously deployed to remove heavy metals from water, the utilization of biodegradable polymer blend foams filled with carbonaceous materials has emerged as an environmentally friendly and sustainable strategy. Biodegradable polymers refer to polymers that can break down in natural environments, in the action of enzymes from microorganisms, giving off harmless products. They are classified into natural and synthetic types. Natural biodegradable polymers, termed biopolymers, are derived from natural sources or occur naturally, and their examples include polysaccharides, cellulose and starch. Synthetic biodegradable polymers are manmade polymers from renewable resources like starch, corn, and sugar beet, designed to naturally break through biological processes [9, 10]. Commonly utilized biodegradable polymers, usually in blended form, include poly(lactic acid) (PLA), poly(ε – caprolactone) (PCL), poly(butyl succinate) (PBS), and poly(3-hydroxybutyrate-co-3-hydroxyvalerate) (PHBV), amongst others [8, 9].

Blending two or more synthetic biodegradable polymers offers more tailorability and fine tuning towards the desired properties like the removal of heavy metals from water. However, most biodegradable polymer blends are inherently phase separated and immiscible, affecting their morphological, thermal, and mechanical properties and, ergo, optimal performance in applications [11]. Due to this, various research has looked at incorporating numerous carbonaceous materials like biochar, carbon nanotubes (CNTs), graphene, graphene oxide (GO), and reduced graphene oxide (rGO) into the matrices of biodegradable polymer blends to improve the said properties [12, 13]. Most carbonaceous materials are derived from renewable resources like biomass, making them suitable for forming composites with biodegradable polymers for environmental remediation purposes. These multifunctional composites would be processed into foams to leverage the resultant high porosity, tuneable morphology, and enhanced adsorption capacities of metal ions from water [14, 15]. The exploration of the synergistic effects between carbonaceous materials and biodegradable polymer blend foams is an emerging research niche, especially for water treatment purposes. These composite foams can effectively adsorb heavy metals from water due to the abundance of oxygen containing functional groups on fillers like graphene oxide, as well as on most biodegradable polymers. These functional groups are capable of chemically complexing the heavy metal ions, while the polar and porous nature of the composite foams creates sites for physical adsorption of the metal ions. Moreover, the large surface area of most carbonaceous materials makes for a high number of adsorption sites, and the foamy nature of the materials increases the strength to weight ratio, forming the so-called super composites.

Despite growing research in the development of biodegradable polymer blend foams filled with carbonaceous materials for the removal of heavy metals in water, there exists a need to comprehensively synthesize and analyse the adsorption efficiencies and capacities, design strategies, structure-property-function relationships, and adsorption mechanisms challenges in this regard. The present systematic review protocol aims to consolidate current knowledge and identify research gaps to guide future development of sustainable adsorbent technologies for heavy metal removal. Consolidating existing evidence will be particularly useful to municipalities in developing countries like South Africa that can use this information to build upon and improve the water and sanitation conditions of the communities.

Stakeholder engagement will form an integral component of the present systematic review. The guidelines by the collaboration for Environmental Evidence (CEE) will inform the consultation of relevant stakeholders to enhance relevance, contextual validity, and applicability of findings, during the development and conduct of the review. These stakeholders will include, officials of municipal water and sanitation, particularly from South African local municipalities with aging or under sourced water treatment infrastructure, Environmental engineers and water treatment practitioners, polymer scientists and materials engineers who work on sustainable water remediation technologies, policy advisors as well as environmental consultants. Initial consultations have informed the framing of the primary question, in particular, the adsorption capacity (mg/g) and removal efficiency (%) as decision-relevant metrics for the implementation of treatment. Stakeholder input will also guide the interpretation of findings, especially to assess feasibility, scalability, and applicability in the context of resource limitedness. Engagement will commence via structured informal discussions, correspondence by email, and brief consultation meetings where feasible. Stakeholders will not influence inclusion decisions, risk of bias assessment, or data extraction processes. They role will strictly be advisory and contextual, to ensure that the synthesis addresses practical water treatment challenges and is in support of evidence-informed decision making. All stakeholder contributions shall be transparently documented in the final review.

## Objective of the review

The review’s primary objective is the systematic evaluation and synthesis of evidence on the following:

1. The efficiency of heavy metal removal in water through adsorption capacities and percentage efficiencies.
2. The strategies and methods utilized in designing and fabricating biodegradable polymer blend foams filled with carbonaceous materials for the adsorption of heavy metals from water.
3. The influence of the resultant polymer composite morphological, structural, thermal, thermos-mechanical, and mechanical properties on adsorption efficiency.
4. Adsorption kinetic and isotherm models, as well as mechanisms that give an in-depth description of the adsorption process.
5. Current challenges and future directions of research, including the inclusion of AI-guided design approaches for optimization.

### Primary question

What is the adsorption efficiency and capacity of biodegradable polymer blend foams filled with carbonaceous materials in the removal of heavy metals from water?

### Secondary questions

1. What are commonly used biodegradable polymers and carbonaceous materials to form the foams?
2. Which blending and foaming techniques are utilized to form the composite foams?
3. How do blend and composite properties like morphology, structural, thermal, thermo-mechanical, and mechanical properties relate to the adsorptive performance of the composite foams?
4. Which kinetics, isotherms and mechanisms have been used by study authors to best describe the adsorption process?

### Components of the question

#### Population

Any type of water body contaminated by heavy metal pollutants.

#### Intervention

The usage of biodegradable polymer blend/carbonaceous material foam composites to adsorb the heavy metals from water.

#### Comparator

Control with no intervention (i.e., untreated water reporting baseline heavy metal concentrations (mg/L) before adsorption to enable calculation of removal efficiency (%)) or water treated with other adsorbents. Controls without reported baseline concentrations remain eligible if absolute adsorption capacity (mg/g) is provided.

#### Outcome

Adsorption capacity (mg/g) and efficiency (%) of composite foams in removing heavy metals from water.

## Methods

The systematic review and the present protocol will be in alignment with the guidelines by the Collaboration for Environmental Evidence (CEE) [16] and the ROSES checklist (Additional file 1).

### Searching for articles

The review will search through and retrieve information from both grey and peer-reviewed sources, like journal articles, books, book chapters, conference papers and reports, that include laboratory and pilot scale investigations. Literature reviews and theoretical studies will not be considered but may be utilized to locate additional references.

### Search strategy and language

The search will be conducted in English for published peer reviewed articles, reports, case studies, books, and theses. The search string will use terms to search for the aspects of the intervention and the population, and these will be combined using Boolean operators “AND” and/or “OR”. There will generally be no restriction on the publication dates to allow for the capturing of early and recent advancements in water treatment techniques. The final search strings presented below will be implemented in the respective bibliographic sources. The development (“polymer blend foam*” OR “biodegradable polymer blend foam*” OR “biodegradable polymeric composite foam*” OR “biobased polymer foam*” OR “biodegradable polymer foam*”) AND (“graphene oxide” OR “reduced graphene oxide” OR “graphene” OR “carbon nanotube*” OR “multiwalled carbon nanotube*” OR “CNT*” OR “biochar” OR “graphite” OR “graphitic oxide”) AND (“heavy metal*” OR “lead” OR “Pb” OR “copper” OR “Cu” OR “cadmium” OR “Cd” OR “chromium” OR “Cr”) AND (“adsorption” OR “adsorbent*” OR “water treat*” OR “water purif*” OR “water remed*” OR “water decontamination”).

The search strings will be adapted to match the specific syntax and field tags required by each bibliographic source (e.g., TS = in Web of Science, TITLE-ABS-KEY in Scopus, and Title/Abstract fields in PubMed/MEDLINE).

### Validation of the comprehensiveness of the search

To ensure that the search strategy is sufficiently comprehensive, the final search string was tested against a benchmark list of known relevant studies identified during the initial scoping phase of the review. This benchmarking approach follows the recommendations of the Collaboration for Environmental Evidence (CEE) guidelines for systematic reviews.

A benchmark list of 15 relevant articles on biodegradable polymer foams and polymer–carbonaceous composite materials used for heavy-metal adsorption from water was compiled from preliminary scoping searches and expert knowledge within the research team. These studies represent key publications in the field and cover a range of polymer systems, carbonaceous fillers, and adsorption applications relevant to the review question.

The final search string was tested in Scopus and the Web of Science Core Collection, and the retrieved results were examined to determine whether all benchmark studies were captured. Initial testing indicated that several relevant studies were not retrieved due to variations in terminology used for polymer foams and carbonaceous materials. The search string was therefore refined by incorporating additional synonyms and truncation operators (e.g., “foam*”, “nanotube*”, “adsorbent*”, and “heavy metal*”) to improve retrieval sensitivity.

Following refinement, the final search string successfully retrieved all studies included in the benchmark list, indicating high search sensitivity and comprehensiveness. The finalised search string is therefore expected to capture most of the relevant literature relating to biodegradable polymer blend foams filled with carbonaceous materials for heavy metal adsorption from water.

The full benchmark article list used to test the search string, along with details of the search string development process, is provided in Additional File 2.

## Bibliographic and grey literature sources

The following bibliographic sources will be searched to identify peer-reviewed literature relevant to the review question. Searches will be conducted using structured Boolean search strings combining terms related to biodegradable polymer foams, carbonaceous materials, heavy metal contamination, and adsorption processes. Search strings will be adapted where necessary to match the syntax requirements of each bibliographic source.

### Web of science core collection

The Web of Science Core Collection will be searched using the Topic field (TS =), which includes title, abstract, and author keywords. The following indices will be searched:

- Science Citation Index Expanded (SCIE).
- Social Sciences Citation Index (SSCI).
- Conference Proceedings Citation Index – Science (CPCI-S).
- Emerging Sources Citation Index (ESCI).

Search string:

#### TS =

((“polymer blend foam*” OR “biodegradable polymer blend foam*” OR “biodegradable polymeric composite foam*” OR “biobased polymer foam*” OR “biodegradable polymer foam*”) AND (“graphene oxide” OR “reduced graphene oxide” OR “graphene” OR “carbon nanotube*” OR “multiwalled carbon nanotube*” OR “CNT*” OR “biochar” OR “graphite” OR “graphitic oxide”) AND (“heavy metal*” OR “lead” OR “Pb” OR “copper” OR “Cu” OR “cadmium” OR “Cd” OR “chromium” OR “Cr”) AND (“adsorption” OR “adsorbent*” OR “water treat*” OR “water purif*” OR “water remed*” OR “water decontamination”))

#### Scopus

Scopus will be searched using the TITLE-ABS-KEY field to capture terms appearing in article titles, abstracts, and author keywords.

Search string:

#### TITLE-ABS-KEY

((“polymer blend foam*” OR “biodegradable polymer blend foam*” OR “biodegradable polymeric composite foam*” OR “biobased polymer foam*” OR “biodegradable polymer foam*”) AND (“graphene oxide” OR “reduced graphene oxide” OR “graphene” OR “carbon nanotube*” OR “multiwalled carbon nanotube*” OR “CNT*” OR “biochar” OR “graphite” OR “graphitic oxide”) AND (“heavy metal*” OR “lead” OR “Pb” OR “copper” OR “Cu” OR “cadmium” OR “Cd” OR “chromium” OR “Cr”) AND (“adsorption” OR “adsorbent*” OR “water treat*” OR “water purif*” OR “water remed*” OR “water decontamination”))

#### PubMed/MEDLINE

PubMed will be searched using the title and abstract fields to identify literature on environmental remediation and adsorption technologies indexed in biomedical and environmental health research.

Search string:

((“polymer blend foam*” OR “biodegradable polymer foam*” OR “biodegradable polymer composite foam*”) AND (“graphene oxide” OR “reduced graphene oxide” OR “graphene” OR “carbon nanotube*” OR “biochar”) AND (“heavy metals” OR “lead” OR “copper” OR “cadmium” OR “chromium”) AND (“adsorption” OR “adsorbent*” OR “water treatment” OR “water purification” OR “water remediation”).

#### Grey literature sources

To minimise publication bias and capture relevant non-peer-reviewed evidence, grey literature will also be searched. This includes theses, institutional reports, and publications from international organisations involved in water quality and environmental remediation.

#### Google Scholar

Google Scholar will be searched using a simplified search string due to platform character limitations. The search will be conducted using the Advanced Search function and the first 200 results sorted by relevance will be screened.

Search string:

“polymer foam” adsorption “heavy metals” graphene OR biochar OR “carbon nanotubes”.

#### Open Access Theses and Dissertations (OATD)

The Open Access Theses and Dissertations repository will be searched for postgraduate theses reporting experimental research on biodegradable polymer composites and heavy-metal adsorption from water.

Search keywords:

biodegradable polymer foam, heavy metal adsorption.

#### Institutional and international organisation websites

Relevant reports and technical documents relating to water contamination and remediation will be searched from the following organisations:

1. United Nations Water (UN-Water) (https://www.unwater.org).
2. UNESCO World Water Assessment Programme (WWAP) (https://www.unesco.org/en/wwap).
3. World Resources Institute (WRI) (https://www.wri.org).

Searches within these websites will use combinations of the following keywords: heavy metal removal, water treatment adsorption, polymer adsorbent, graphene oxide adsorption, biochar water remediation.

#### Citation searching

To identify additional relevant studies that may not be captured through database searches, forward and backward citation searching will be conducted. Backward citation searching will involve examining the reference lists of included studies, while forward citation searching will be performed using the Google Scholar “Cited by” function to identify newer studies citing the included articles.

#### Calls for evidence through research networks

To ensure that potentially relevant studies not indexed in bibliographic databases are identified, a call for evidence will be issued through professional and academic research networks. These platforms will not be used as primary search sources but rather as a mechanism to invite researchers and practitioners to share relevant studies, reports, or unpublished research related to the review topic.

The following academic networking platforms will be used to disseminate the call for evidence:

1. ResearchGate (https://www.researchgate.net).
2. Academia.edu (https://www.academia.edu).

The call for evidence will briefly describe the scope of the systematic review and invite researchers in polymer science, materials science, environmental engineering, and water treatment to share relevant publications, reports, theses, or ongoing studies on biodegradable polymer composite foams and heavy metal adsorption from water. Posts will include keywords related to the review topic, including: biodegradable polymer foams, polymer composite adsorbents, graphene oxide composites, carbon nanotube composites, biochar composites, heavy metal adsorption, water remediation.

Any studies identified through these calls will be screened using the same eligibility criteria and screening procedures applied to studies retrieved from bibliographic and grey literature searches. Social media platforms such as Facebook, X (formerly Twitter), TikTok, and YouTube will not be used as literature search sources but may be used only to disseminate the call for evidence where appropriate.

### Article screening and eligibility criteria

#### Screening process

The screening process will follow the reporting standards recommended by the Collaboration for Environmental Evidence (CEE) and the ROSES (Reporting Standards for Systematic Evidence Syntheses) framework. All records identified through the database and grey literature searches will first be exported into a reference management software package, where duplicate records will be removed prior to screening. Screening will occur in two stages: title and abstract screening, followed by full-text screening. During title and abstract screening, two reviewers (Lesia S. Mokoena and Julia P. Mofokeng) will independently screen all retrieved records against the predefined eligibility criteria. Studies that clearly do not meet the eligibility criteria will be excluded at this stage. If there is uncertainty about the relevance of a study based solely on its title and abstract, it will be retained for full-text screening.

All studies considered potentially relevant will proceed to full-text screening, where the same two reviewers will independently assess the full texts against the eligibility criteria. Any disagreements between reviewers during either screening stage will be resolved through discussion and consensus. If consensus cannot be reached, a third reviewer (Pennie P. Mokolokolo) will act as an adjudicator. To ensure consistent application of the eligibility criteria, a calibration exercise will be conducted prior to the formal screening process. A random subset of retrieved records will be independently screened by both reviewers, and the screening criteria will be refined if necessary to ensure consistent interpretation. A record of all excluded studies at the full-text screening stage will be maintained, together with the specific reasons for exclusion. These will be reported in the final review in accordance with ROSES reporting standards.

#### Eligibility criteria

To be included in the systematic review, studies must fulfil the following criteria:

##### Eligible populations

Studies must report on any type of water bodies contaminated by heavy metal pollutants.

##### Eligible interventions

Studies must include the utilisation of one or more common biodegradable polymer blend foams, PLA/PCL, PLA/PHBV, PLA/PBS, PCL/PHBV, PCL/PBS, PHBV/PBS, and others, filled with carbonaceous fillers like GO, biochar, CNTs, rGO, to remove heavy metal ions from water.

##### Eligible comparators

Studies may report comparisons between biodegradable polymer composite foams and untreated water or water treated with conventional adsorbents such as activated carbon, zeolites, or other polymeric adsorbents. Studies without a direct comparator will also be eligible if they report quantitative adsorption performance metrics, including adsorption capacity (mg/g) and/or removal efficiency (%). In such cases, outcomes will be assessed based on the reported adsorption performance under defined experimental conditions (e.g., initial metal ion concentration, pH, contact time, and temperature), and comparisons will be conducted across studies during the evidence synthesis rather than against a single predefined benchmark.

##### Eligible outcomes

Studies should report mainly on the adsorption capacity (mg/g) and effectiveness (% removal) of polymer composite foam adsorbents.

##### Eligible language

Explored studies should be published in the English language.

### Study validity assessment

The methodological validity of each included study will be critically appraised using a structured checklist adapted from the Collaboration for Environmental Evidence (CEE) guidelines for systematic reviews. This checklist will assess internal validity (e.g., study design and control of confounding factors), external validity (e.g., relevance and generalizability to other contexts), and reporting transparency (e.g., completeness and clarity of methods and results). The developed checklist will cover the following domains:

1. **Experimental design and methodology**
  The methodological validity of each included study will be evaluated against predefined criteria for the clarity and rigour of the experimental design and methodology. These criteria will be assessed using operational definitions to ensure consistent interpretation across reviewers.

2. **Clear description of materials and fabrication methods**
  Studies must provide sufficient detail regarding the materials and fabrication procedures used to produce the biodegradable polymer composite foams. This includes reporting the types of polymers used in the blend, the type and loading of carbonaceous fillers, and the processing or foaming technique applied. A description will be considered adequate if the reported methods would allow an independent researcher to prepare a comparable material.

3. **Appropriate controls**
  Studies should include an appropriate control condition to enable interpretation of adsorption performance. Acceptable controls include untreated water samples, adsorption experiments conducted without the composite foam adsorbent, or comparisons with conventional adsorbents such as activated carbon or similar materials.

4. **Replicable experimental procedures**
  Experimental procedures must be described in sufficient detail to allow replication. This includes reporting key adsorption testing conditions such as pH, temperature, contact time, agitation conditions, initial metal ion concentration, and adsorbent dosage.

5. **Adequate reporting of experimental replicates**
  Studies should report whether adsorption experiments were conducted with replicates (e.g., duplicates or triplicates) or provide statistical measures, such as standard deviations, standard errors, or confidence intervals. This information will be used as an indicator of the reliability of the reported results.

6. **Description of adsorption testing conditions**
  Studies must clearly report the experimental conditions under which adsorption tests were performed, including pH, temperature, contact time, adsorbent dosage, and initial metal ion concentrations. This information is necessary for interpreting adsorption performance and comparing results across studies.

7. **Use of established characterisation techniques**
  Studies should employ appropriate and commonly accepted characterisation techniques to evaluate the structure and properties of the composite foams. These may include microscopy techniques (e.g., scanning electron microscopy), spectroscopic methods (e.g., Fourier transform infrared spectroscopy), thermal analysis (e.g., thermogravimetric analysis), and mechanical or surface characterisation methods.

8. **Transparent reporting of adsorption modelling**
  Where adsorption kinetics or isotherm models are applied, studies should clearly report the modelling approach used, including the model equations applied, the estimated parameters, and measures of model fit such as regression coefficients.

### Reproducibility and transparency

- Studies should provide a detailed reporting of all the compositions of materials used and the processing parameters.
- Where possible, studies should provide raw and supplementary data.
- Studies should provide a clear justification of the selection of participant materials as well as targeted heavy metals.

### Risk of bias

- Studies should clearly account for potential confounding parameters, like the presence of competing ions and the complexity of water matrices.
- Studies should avoid presenting and reporting on only selected outcomes but cover the whole spectrum of the conducted work.
- Studies should disclose any funding sources or conflicting interests capable of altering the results.

The methodological validity of each included study will be critically appraised using a structured checklist adapted from the Collaboration for Environmental Evidence (CEE) guidelines for systematic reviews. The validity assessment will evaluate potential sources of bias in experimental design, methodological reporting, and data transparency. Rather than assigning numerical scores, studies will be categorised into three levels of methodological validity based on the overall assessment of the criteria described above:

### High validity

Studies classified as high validity will demonstrate clear and detailed descriptions of materials and experimental procedures, appropriate controls or baseline measurements, well-reported adsorption testing conditions, and transparent reporting of results and analytical methods. These studies provide a high level of confidence in the reliability and reproducibility of the reported findings.

### Medium validity

Studies classified as medium validity will meet most validity criteria but may include minor methodological limitations or incomplete reporting of some experimental parameters. Although these studies remain useful for evidence synthesis, their findings will be interpreted with moderate caution.

### Low validity

Studies classified as low validity will exhibit substantial methodological limitations, such as insufficient description of experimental procedures, inadequate reporting of key adsorption conditions, lack of experimental replicates, or unclear analytical methods. These studies will be retained for transparency but will be considered with caution during evidence synthesis.

Two reviewers will independently assess the methodological validity of each included study. Prior to the full assessment, a calibration exercise will be conducted on a subset of studies to ensure consistent interpretation of the validity criteria. Disagreements between reviewers will be resolved through discussion and, where necessary, consultation with a third reviewer. The results of the validity assessment will be reported transparently in the final review and will inform the interpretation of the evidence during the synthesis stage. Sensitivity analyses may also be conducted to evaluate the influence of studies with lower methodological validity on the overall findings.

### Data coding and extraction strategy

Data will be systematically extracted from all included studies using a pre-designed, pilot-tested data extraction form in Microsoft Excel. The data will be extracted in duplicate by two independent reviewers to minimise errors and ensure completeness. Any arising discrepancies shall be resolved by consensus or through consultation with a third reviewer. For missing or unclear data (e.g., unreported adsorption capacities or processing conditions), corresponding authors will be contacted via email (using institutional addresses from publications) with a standardised request form listing the specific data required. Two attempts will be made over 4 weeks; non-responses will be documented as ‘unavailable’. Extracted data from author responses will be confirmed by cross-checking with published methods/figures. All extracted data records (Excel sheets with study ID, extracted variables, reviewer initials, missing data status) will be made available as supplementary files (additional file 2) with the final review, enabling verification and meta-analysis replication.​The extraction will focus on key information pertaining to *Study identification and context*, *Materials and synthesis details*, *Characterization of composite foams*, *Adsorption performance*, and *Additional information*. The specific criteria are explained below.

#### Study identification and context

- Author (s), year of publication, and country of study.
- Type of study: laboratory scale, pilot scale, or field trials.
- The source and type of water tested (e.g., wastewater, potable water).

#### Materials and synthesis details

- Types and proportions of biodegradable polymers in blends.
- Types and levels of loading of the carbonaceous fillers (e.g., GO, biochar, carbon nanotubes) in the polymer matrices.
- The methods applied to fabricate the polymeric foams (e.g., solvent-induced, supercritical CO_2_ foaming).
- The conditions of processing, like temperature and pressure.

#### Characterisation of composite foams

- Morphological properties (particle size, pore size, surface area, phase separation, miscibility, chemical/physical interactions).
- Thermal and thermomechanical properties (glass transition temperatures, melting temperatures, crystallisation, enthalpies, thermal degradation).
- Mechanical properties (Tensile strength, impact resistance, elasticity).

#### Adsorption performance

- Heavy metal(s) targeted (Cu, Pb, Cr, Cd) and concentrations tested.
- The conditions of adsorption (pH, contact time, initial metal ion concentration, temperature).
- Adsorption capacity (mg/g) and the removal efficiency (%).
- Adsorption kinetics and at least two isotherm modelling parameters and their fit metrics.
- Selectivity toward the adsorption of specific metal ions over others (competing ions).

#### Additional information

- Sources of funding and potential conflicting interests.
- Authors noted limitations, challenges and recommendations.
- Any usage or mention of AI or computational approaches.

### Potential effect modifiers/reasons for heterogeneity

The listed key effect modifiers and sources of heterogeneity have been identified for coding and consideration for this review. These variables are considered to most likely influence the reported adsorption performance of biodegradable polymer blend foams filled with carbonaceous materials for the removal of heavy metal ions from water. The list was compiled through an initial scoping review of available literature, iterative consultation of relevant reviews, the expertise of the review team, and feedback from polymer science and environmental engineering experts consulted during the development of the protocol. The list of modifiers to be considered is as follows:

1. Adsorption kinetics and isotherms, metal ion selectivity, recyclability in terms of the number of cycles and retention of adsorption capacity, composite properties (morphology, structural, thermal, thermo-mechanical, and mechanical) relating to application.
2. Type of biodegradable polymer blend (e.g., PLA/PCL, PLA/PHBV, PCL/PBS, PCL/PHBV, etc.).
3. Type and loading of carbonaceous fillers (e.g., graphene oxide, reduced graphene oxide, biochar, at different loading weight percentages like 0.5, 1, 3, 5, 7, 10 wt% etc.).
4. The method used in fabricating foams (e.g., supercritical CO_2_ foaming, solvent casting, thermal melt-mixing, etc.).
5. Adsorption testing conditions (e.g., pH, contact time, temperature, heavy metal ion concentrations, co-existing/competing ions).
6. Scale of study, whether laboratory, pilot, or field.
7. Geographical region/water source, focus on country, region, and whether the contaminated water is synthetic or real (from municipal, industrial, etc.).

It is important to note that at the protocol stage, this list might not be exhaustive, and more effect modifiers might be found during the review process.

### Data synthesis and presentation

To give a thorough and transparent synthesis of the collected data, both narrative and quantitative data will be used. The presented dual approach accommodates heterogeneity in study designs, outcome measures, and reporting in the literature on polymer blend foams filled with carbonaceous adsorbents for heavy metal remediation.

1. **Narrative synthesis**
  An attempt at a narrative synthesis of all collected studies will take place, regardless of data type or the feasibility of quantitative pooling. This synthesis will involve systematically tabulating and describing the characteristics, interventions, materials, context, outcomes, and validity ratings of each study. Data coding and extraction forms will be used to generate summary tables and figures that highlight key details, including polymer/filler types, fabrication methods, outcome measures, performance metrics, and study quality. Major results, such as adsorption capacities and efficiencies, will be visualised using descriptive statistics (medians, ranges, means) alongside supporting text to provide context for the evidence.

2. **Quantitative synthesis (where feasible)**
  Where sufficient, relatively homogenous quantitative data are available (e.g., adsorption capacity in mg/g for similar adsorbate/adsorbent types under comparable conditions), meta-analysis will be conducted. Effect sizes, like raw mean differences in adsorption capacities or removal efficiencies, will be calculated or extracted for each study. Weighted meta-analyses, using either fixed-effect or random-effects models, depending on heterogeneity as assessed by the I^2^ statistic, will be used to aggregate results for each outcome.

### Effect size selection by comparator type

Effect sizes vary by comparator and outcome, ensuring all studies contribute meaningfully:

- No intervention (before-after studies): Primary effect size = adsorption capacity (q_e_ mg/g) as raw mean (universally reported, directly comparable).
  $$\:\mathrm{\%}\:\mathrm{R}\mathrm{e}\mathrm{m}\mathrm{o}\mathrm{v}\mathrm{a}\mathrm{l}=\:\frac{{\mathrm{C}}_{0}-{\mathrm{C}}_{\mathrm{e}}}{{\mathrm{C}}_{0}}\times\:100$$
  where C_0_ = baseline concentration (mg/L).

- Other adsorbents: Mean difference (MD) in adsorption capacity (mg/g) between foam composites vs. conventional adsorbents; or standardised mean difference (SMD) if units differ; relative % efficiency difference.
- Single-arm studies (no comparator): Adsorption capacity (mg/g) contributes to narrative synthesis plus descriptive statistics (medians, ranges by polymer/filler type); excluded from comparative meta-analysis but included in overall capacity distributions.

All effect sizes will be extracted per study design (Additional File 2). Fixed/random-effects models per I² heterogeneity; sensitivity analyses exclude low-quality studies.

## Supplementary Information

Supplementary Material 1. Additional file 1: ROSES checklist. Completed ROSES (Reporting Standards for Systematic Evidence Syntheses) protocol checklist used to guide the reporting structure of this systematic review protocol. Additional file 2: Data coding and extraction form. Microsoft Excel template used for systematic data extraction and coding from included studies. The file includes fields for study identification, material composition, synthesis methods, characterisation results, adsorption performance metrics, and additional contextual information relevant to the review. Additional file 3: Study exclusion log. Microsoft Excel file documenting full-text articles assessed for eligibility but excluded from the review, along with the specific reasons for exclusion. This file ensures transparency and reproducibility of the study selection process. Additional file 4: Search validation and benchmarking framework. Microsoft Excel file describing the validation process used to assess the comprehensiveness of the search strategy, including benchmark studies used to test the final search strings and documentation of the search sensitivity assessment.

## Data Availability

No datasets were generated or analysed during the current study.

## References

[1] Tchounwou PB, Yedjou CG, Patlolla AK, Sutton DJ. Heavy metal toxicity and the environment. Exp Suppl. 2012;101:133–64. 22945569 10.1007/978-3-7643-8340-4_6 PMC4144270

[2] Jaishankar M, Tseten T, Anbalagan N, Matthew BB, Beeregowda KN. Toxicity, mechanism and health effects of some heavy metals. Interdiscip Toxicol. 2014;7:60–72. 26109881 10.2478/intox-2014-0009 PMC4427717

[3] Ali H, Khan E, Ilahi I. Environmental chemistry and ecotoxicology of hazardous heavy metals: environmental persistence, toxicity, and bioaccumulation. J Chem. 2019;2019:6730305.

[4] Fu F, Wang Q. Removal of heavy metal ions from wastewaters: a review. J Environ Manage. 2011;92:407–18. 21138785 10.1016/j.jenvman.2010.11.011

[5] Barakat MA. New trends in removing heavy metals from industrial wastewater. Arab J Chem. 2011;4:361–77.

[6] Olunyika OA, Patel AV, Shah BA, Bagia MI. Microwave and fusion techniques for the synthesis of mesoporous zeolitic composite adsorbents from bagasse fly ash: sorption of p-nitroaniline and nitrobenzene. Appl Water Sci. 2020;10:1–20.

[7] United Nations. Transforming our world: The 2030 Agenda for Sustainable Development. 2015. https://sdgs.un.org/2030agenda. Accessed 03 July 2025.

[8] World Health Organization. Guidelines for drinking-water quality: Fourth edition incorporating the first addendum. 2017. https://www.who.int/publications/i/item/9789241549950. Accessed 03 July 2025. 28759192

[9] Thakur VK, Thakur MK, Raghavan P, Kessler MR. Progress in green polymer composites from lignin for multifunctional applications: a review. ACS Sustain Chem Eng. 2018;6:9340–60.

[10] Niaounakis M. Biopolymers: applications and trends. Norwich NY: William Andrew Publishing; 2015.

[11] W Z, Zhang C, Zhu Y, Lu Z, Liu H, Xu B, et al. Visualisation of macrophase separation and transformation in immiscible polymer blends. CCS Chem. 2023;5:718–28.

[12] Tan X, Li Y, Zeng G, Wang X, Hu X, Gu Y, Yang Z. Application of biochar for the removal of pollutants from aqueous solutions. Chemosphere. 2015;125:70–85. 25618190 10.1016/j.chemosphere.2014.12.058

[13] Mokoena LS, Mofokeng JP. Morphology and thermal properties of poly(lactic acid)/poly(3-hydroxybutyrate-co-3-hydroxyvalerate)/graphene oxide polymeric composites. Polym Eng Sci. 2024;64:5329–50.

[14] Gonçalves LFFF, Reis RL, Fernandes EM. Forefront research of foaming strategies on biodegradable polymers and their composites by thermal or melt-based processing technologies: advances and perspectives. Polymers. 2024;16:1286. 38732755 10.3390/polym16091286 PMC11085284

[15] Gaidhani A, Tribe L, Charpentier P. Polystyrene carbon composite foam with enhanced insulation and fire retardancy for a sustainable future: critical review. J Cell Plast. 2023;59:419–45.

[16] Pullin AS, Frampton GK, Livoreil B, Petrokofsky G, Edelstein KE, Collaboration for environmental evidence. Guidelines and standards for evidence synthesis in environmental management. Version 5.1. 2022. www.environmentalevidence.org/information-for-authors. Accessed 17 July 2025.

