# Supplementary material for: What is the adsorption efficiency and capacity of biodegradable polymer blend foams filled with carbonaceous materials in the removal of heavy metals from water? A systematic review protocol
Source: Environ Evid. 2026 Mar 29;15:4. doi: 10.1186/s13750-026-00384-x (PMC13151128; doi:10.1186/s13750-026-00384-x)
Supplement: Supplementary file 1 — Supplementary Material 1. Additional file 1: ROSES checklist. Completed ROSES (Reporting Standards for Systematic Evidence Syntheses) protocol checklist used to guide the reporting structure of this systematic review protocol. Additional file 2: Data coding and extraction form. Microsoft Excel template used for systematic data extraction and coding from included studies. The file includes fields for study identification, material composition, synthesis methods, characterisation results, adsorption performance metrics, and additional contextual information relevant to the review. Additional file 3: Study exclusion log. Microsoft Excel file documenting full-text articles assessed for eligibility but excluded from the review, along with the specific reasons for exclusion. This file ensures transparency and reproducibility of the study selection process. Additional file 4: Search validation and benchmarking framework. Microsoft Excel file describing the validation process used to assess the comprehensiveness of the search strategy, including benchmark studies used to test the final search strings and documentation of the search sensitivity assessment. [file ROSES for Systematic Review Protocols_LS Mokoena.pdf › ROSES for Systematic Review Protocols_LS Mokoena.pdf]

ROSES for Systematic Review Protocols. Version 1.0

November 2017
Neal R Haddaway, Biljana Macura, Paul Whaley, and Andrew Pullin

Column descriptions

Section/sub-section - The sections or sub-sections listed below correspond to sections in CEE systematic review and map protocols and final reports: please see

Topic - the section or sub-section is further broken down for the purposes of clarity.

Description/Further explanation - Details of what is needed in each section or sub-section are provided, along with practical advice and links to relevant guidanc

Checklist/Meta-data - Checklist items MUST be completed. Meta-data items correspond to information that should be reported as data or short descriptions th

Author response - authors should select or write a response for meta-data, or select 'yes' or 'no' for checklist items. Further details may be provided in Commer

Comments - authors may supplement their response with a brief comment.

Please cite as: Haddaway NR, Macura B, Whaley P, and Pullin AS. 2017. ROSES for Systematic Review Protocols. Version 1.0. DOI: 10.6084/m9.figshare.5897269.

at will be used to populate the Summary Record and Flow Diagram. Any meta-data items where stages were not performed (e.g. grey literature searching), this

should be stated (e.g. ‘Not performed’).

Item number	Section / sub-section	Topic
1	Title	Title
2	Type of review	Type of review
3	Authors contacts	Authors contacts
4	Abstract	Structured summary
5	Background	Background
6	Stakeholder engagement	Stakeholder engagement
7	Objective of the review	Objective
8		Definitions of the question
	Methods	
9	Searches	Search strategy
10		Search string
11		Languages – bibliographic
12		Languages – grey literature
13		Bibliographic databases
14		Web – based search engines
15		Organisational websites
16		Estimating the comprehensiveness
17		Search update
18	Article screening and study	Screening strategy
19		Consistency checking
20		Inclusion criteria
21		Reasons for exclusion
22	Critical appraisal	Critical appraisal
23		Critical appraisal strategy
24		Consistency checking
25	Data extraction	Meta-data extraction and coding
26		Data extraction strategy
27		Approaches to missing data
28		Consistency checking
29	Potential effect modifiers/reasons	Potential effect modifiers/reasons
30	Data synthesis and presentation	Data synthesis and presentation

31		Narrative synthesis strategy
32		Quantitative synthesis strategy
33		Qualitative synthesis strategy
34		Other synthesis strategies
35		Assessment of risk of publication
36		Knowledge gap identification
37		Demonstrating procedural
38	Declarations	Competing interests

References

[1] Bayliss, H.R., Haddaway, N.R., Eales, J., Frampton, G.K. and James, K.L., 2016. Updating and a

[2] Haddaway, N.R., Kohl, C., da Silva, N.R., Schiemann, J., Spök, A., Stewart, R., Sweet, J.B. and W

[3] Collaboration for Environmental Evidence. 2018. Guidelines and Standards for Evidence synth

[4] Leeds Institute of Health Sciences. https://medhealth.leeds.ac.uk/info/639/information_spec

Description

The title must indicate that it is a systematic review protocol, and must
Select one of the following types of review: systematic review, systematic
The full names, institutional addresses, and email addresses for all authors
Abstract must not exceed 350 words and must include two sections 1)
Describe the rationale for the review in the context of what is already
The planned/actual role of stakeholders throughout the review process
Describe the primary question and secondary questions (when applicable).
Break down and summarise question key elements e.g. population,

Detail the planned search strategy to be used, including: database names
Provide Boolean-style full search string and state the platform for which
List languages to be used in bibliographic database searches.
List languages to be used in organizational websites searches and web-
Provide the number of bibliographic databases to be searched.
Provide the number of web – based search engines to be searched.
Provide the number of organisational websites to be searched.
Describe the process by which the comprehensiveness of the search
Describe any plans to update the searches during the conduct of the
Describe the methodology for screening articles/studies for
Describe clearly the process for checking consistency of decisions including
Describe the inclusion criteria used to assess relevance of identified
State that you will provide a list of articles excluded at full text with
Describe here the method you propose for critical appraisal of study
Describe how the information from critical appraisal will be used in
Describe how repeatability of critical appraisal of study validity will be
Describe the method for meta-data extraction and coding for studies
Describe the method for extraction of qualitative and/or quantitative study
Describe any processes for obtaining and confirming missing or unclear
Describe how repeatability of the meta-data/data extraction process will
Provide a list of and justification for the effect modifiers /reasons for
State the type of synthesis conducted as part of the systematic review

Describe methods to be used for narratively synthesising the evidence base
If data are appropriate for quantitative synthesis, describe planned
Describe methods to be used for synthesising qualitative data and justify
Describe any other approaches to be used for synthesising data or
Describe planned methods for examining the possible influence of
Describe the methods to be used to identify and/or prioritise key
Describe the role of systematic reviewers (who have also authored articles
Describe of any financial or non-financial competing interests that the

mending systematic reviews and systematic maps in environmental management. E
Vilhelm, R., 2017. A framework for stakeholder engagement during systematic revie
hesis in Environmental Management. Version 5.0. www.environmentalevidence.org
cialists/1500/search_concept_tools. Accessed 12/11/2017.

Further explanation	Checklist/Meta-data
The title should normally be the same or very similar to the review	Meta-data
See CEE Guidance on amendments and updates [1]	Meta-data
	Checklist
	Checklist
A theory of change and/or conceptual model can be presented that links	Checklist
	Checklist
The primary question is the main question of the review. Secondary	Checklist
For other question types see [3,4]	Meta-data
Details regarding search strategy testing should be provided.	Checklist
	Meta-data
	Meta-data
	Meta-data
	Meta-data
	Meta-data
	Meta-data
	Checklist
Optional. A search update is good practice if original searches were	Checklist
	Checklist
	Checklist
	Checklist
	Checklist
	Checklist
	Checklist
	Checklist
	Checklist
	Checklist
	Checklist
	Checklist
The list should not be exhaustive but a short list of those variables thought	Checklist
	Meta-data

Vote-counting (tallying of studies based on the direction or significance of	Checklist
Compulsory if appropriate for data	Checklist
Compulsory if appropriate for data	Checklist
Compulsory if appropriate for data	Checklist
For quantitative syntheses this may be done using diagnostic plots or	Checklist
Optional	Checklist
Reviewers who have authored articles to be considered within the review	Checklist
	Checklist

:nvironmental Evidence, 5(1), p.20.
:ws and maps in environmental management. *Environmental Evidence* , *6* (1), p.11.
/information-for-authors.

Author response	Comments
Biodegradable polymer blend foams filled with carbonaceous materials	
systematic review	
Yes	
Yes	
Yes	
Yes	
Yes	
Components of the question	
Yes	
(“ polymer blend foams” OR “biodegradable polymer blend foams” OR	
English	
English	
5	
6	
Yes	
Yes	
Yes	
Yes	
Yes	
Yes	
Yes	
Yes	
Yes	
Yes	
Yes	
Yes	
Yes	
Yes	
Narrative and quantitative	

Yes
Yes
Yes

Yes
Yes
Yes
Yes
